# *Agrobacterium*-Mediated High-Efficiency Genetic Transformation and Genome Editing of Chaling Common Wild Rice (*Oryza rufipogon* Griff.) Using Scutellum Tissue of Embryos in Mature Seeds

**DOI:** 10.3389/fpls.2022.849666

**Published:** 2022-03-24

**Authors:** Zhipan Xiang, Yi Chen, Yan Chen, Lin Zhang, Min Liu, Dandan Mao, Liangbi Chen

**Affiliations:** Hunan Province Key Laboratory of Crop Sterile Germplasm Resource Innovation and Application, College of Life Sciences, Hunan Normal University, Changsha, China

**Keywords:** *Agrobacterium*-mediated genetic transformation, genome editing, scutellum tissue of embryos in mature seeds, Chaling common wild rice, highly efficient transformation system

## Abstract

Genetic transformation is an important strategy for revealing gene function, and it is used extensively in both functional genomics study and molecular breeding of rice. Demand for its application in wild *Oryza* species is rising for their extensive genetic diversity. However, genetic transformation of wild *Oryza* accessions with AA genome using calli induced from scutellum tissue of embryos in mature seeds has not been successfully established. In the present study, we used Chaling common wild rice (CLCWR) (*Oryza rufipogon* Griff.) with AA genome to successfully establish an *Agrobacterium*-mediated genetic transformation system based on scutellum tissue of embryos in mature seeds. The calli from embryos in mature seeds of CLCWR were easy to be induced and regenerated. The callus induction rate and texture were optimum under 2.5 mg/L 2,4-D. The optimal hormone combination used for regeneration was 2 mg/L ZT + 0.1 mg/L NAA. Studies on genetic transformation and genome editing showed that the transformation efficiency was 87–94%, the efficiency of single genome editing and multiplex genome editing were about 60–70% and 20–40%, respectively. Compared with Nipponbare (Nip), CLCWR had higher *Hygromycin*-resistant callus frequency and transformation efficiency. Taken together, our study establishes a highly efficient transformation system for common wild rice with AA genome and provides a good rice material for *de novo* domestication by genome editing in the future.

## HIGHLIGHTS

- High-efficiency genetic transformation and genome editing of Chaling common wild rice.

## Introduction

Rice (*Oryza sativa* L.) is one of the most important crops in the world. Breeding new rice cultivars with strong tolerance to environmental changes is an urgent goal (Wheeler and von Braun, 2013; Pugh et al., 2016). Wild rice can survive well in the wild field due to its strong resistant ability to a lot of adversity stresses, such as cold (Ma Y. et al., 2015; Mao et al., 2015; Jiang et al., 2019), salt (Prusty et al., 2018; Yichie et al., 2018), waterlogging (Kuroha et al., 2018), and drought (Ramachandran and Khan, 1991; Zhang et al., 2014, 2017; Li et al., 2019; Xu et al., 2021). Wild *Oryza* species have extensive genetic diversity, thus enabling a better adaptation to fluctuating environments. Genetic variation is the basis of agricultural improvement, so the utilization of wild *Oryza* species in basic science and breeding is rising. In the future, creating new rice germplasm with excellent characteristics by using wild rice is a powerful way to solve the food problem (Chen et al., 2021). However, wild rice has some adverse traits, such as prostrate growth (Tan et al., 2008), high plant height (Zhang L. et al., 2020), long awn (Hua et al., 2015), seed shattering (Lin et al., 2007), and long heading date (Dai et al., 2012; Li et al., 2018). Traditional cross-breeding cannot eliminate these adverse traits quickly, and it is difficult to obtain the excellent traits of both parents at the same time. With the development of molecular biology, directional modification of relative genes in wild rice using genome editing technology is the rapid and direct way to create new rice germplasm resources (Chen et al., 2019; López-Marqués et al., 2020; Gao, 2021).

At present, *Agrobacterium*-mediated genetic transformation of rice has been generally used in functional genomics study and molecular breeding (Hiei et al., 1994; Zhang et al., 1997; Hiei and Komari, 2008; Ozawa, 2012). Because of the diversity and complexity of the genetic background of wild rice (Stein et al., 2018), its high-efficiency genetic transformation system has not been well established. To date, 21 wild rice species existed in the world, which are divided into 11 genotypes according to their genomic characteristics: AA, BB, CC, BBCC, CCDD, EE, FF, GG, HHJJ, HHKK, and KKLL (Wing et al., 2018). Allotetraploid *Oryza alta* Swallen with CCDD genome from South America, possesses a variety of disease and insect resistances (Mao et al., 1995; Ammiraju et al., 2010). The tissue culture study of Swallen showed that the callus induction rate and regeneration frequencies were only 20 and 10%, respectively (Zhang et al., 2019), suggesting that its callus induction and regeneration ability were extremely low. Polyploid rice 1 (PPR1) from South America is also an allotetraploid wild rice with CCDD genome. Though the callus induction rate and transformation efficiency of PPR1 are higher than that of Nip (*japonica*), its regeneration ability is lower (Yu et al., 2021).

Common wild rice (CWR) with AA genome, which is the immediate ancestral progenitor of cultivated rice in Asia containing various beneficial genes and can be potentially used in rice breeding in the future by molecular modification (Huang et al., 2012; Brozynska et al., 2016). Yuanjiang common wild rice (YJCWR) with AA genome, from Yuanjiang County, Yunnan Province, China, shows strong callus browning resistance caused by oxidative stress (Zhang K. et al., 2020). Recently, genetic transformations of several CWR with AA genome by using immature embryos were studied, and found that the callus induction rate and regeneration efficiency were high. However, its infection efficiency was only 12.5–37.5%, which is lower than that of Nip. In addition, compared with scutellum tissue of embryos in mature seeds which can be conveniently collected and stocked enough mature seeds to run experiments, the operation of callus induction using immature embryosis is more cumbersome (Shrawat and Good, 2011; Shimizu-Sato et al., 2020). Therefore, it is necessary to establish an *Agrobacterium*-mediated high-efficiency genetic transformation system of CWR by using scutellum tissue of embryos in mature seeds.

Chaling common wild rice is one of the common wild rice species with AA genome, originally lives in Chaling County (113° 40′ E, 26° 50′ N), Hunan Province, China (Wang et al., 2020; Xu et al., 2020). CLCWR has larger anthers and a high stigma exposure rate than cultivated rice. More importantly, CLCWR has strong cold tolerance and disease resistance (Xu et al., 2020). Therefore, CLCWR is important genetic rice germplasm, and possesses important value of research and utilization. However, the genetic transformation and genome editing system of CLCWR is not established. Here, we report a successfully established *Agrobacterium*-mediated high-efficient genetic transformation and genome editing systems for CWR with AA genome using scutellum tissue of embryos in mature seeds of CLCWR, which will provide a good rice material for *de novo* domestication for CWR by genome editing in the future.

## Materials and Methods

### Plant Growth and Stress Treatment

Chaling common wild rice, one of the common wild accessions (*Oryza rufipogon* Griff.), originally grows in the ‘Huli’ wetland (113° 40′E, 26°50′N) of Yaoshui Town, Chaling County, Hunan Province, China, with a total area of 40 hectares. CLCWR plants were planted in Changsha City (112° 59′E, 28° 12′N), Hunan Province, China. In the late March every year, the seeds of CLCWR (dormancy broken) were soaked for 24 h and germinated in a 37°C constant temperature incubator for 7 days. The seeds were washed with sterile water once a day. The germinated young seedlings were planted in the soil and cultured in a greenhouse [28°C, 12.5 h light (3,000–5,000 LX)/11.5 h dark] under normal water and fertilizer management. In the middle and late September, CLCWR began to flower. At this time, the whole spike should be covered with a net bag to collect seeds. The mature seeds were harvested and stored at the end of October every year.

For the phenotype analysis of CLCWR mutants under cold treatment, the 30-day plants that grew in soil were treated for 3 days under 4–6°C conditions, then recovered for 7 days. The survival rate was counted and the chlorophyll content of leaves was measured.

### Construction of Genome-Editing and β-Glucuronidase Reporter Vector

Clustered regularly interspaced short palindromic repeat (CRISPR)/Cas9-mediated single genome and multiplex genome editing of CLCWR were generated by using the multiplex genome editing system (provided by Professor Yaoguang Liu, South China Agricultural University). The *9-cis-epoxycarotenoid dioxygenase* genes (*NCED1*, *NCED3*, and *NCED5*) of CLCWR were sequenced according to the corresponding genes sequence of Nip. The genome editing target sites of the three genes were designed according to the website https://blast.ncbi.nlm.nih.gov/Blast.cgi. The editing vectors were constructed according to the previous method (Ma X. et al., 2015). The sgRNAs expression cassettes were generated by using the overlapping PCR method, and then ligated into pYLCRISPR/Cas9 Pubi-H vector by the Golden Gate cloning method.

For the construction of the vector containing the GUS reporter gene, the promoter fragments of *NCED3* and *NCED5* were amplified and ligated into pCAMBIA1301 vector containing *hygromycin phosphotransferase* and β*-glucuronidase* gene, respectively. Primers were listed in Supplementary Table 1.

The genome editing or GUS reporter vectors were then transferred into *Agrobacterium tumefaciens* strain EHA105 by electroporation in an *Escherichia coli* pulser (Bio-Rad).

### Tissue Culture and *Agrobacterium*-Mediated Transformation

The culture medium used in this study was shown in the following tables. Tissue culture by using mature seeds and *Agrobacterium* mediated-transformation methods of Nip referred to the previous protocol (Nishimura et al., 2006). The compositions of the mediums are shown in Supplementary Table 2.

The healthy and plump mature seeds with out glume of CLCWR whose dormancy was eliminated were selected and washed with sterilized water three times. The seeds were disinfected with 75% ethanol for 3 min and shaken with 3% NaClO solution, 180 rpm for 40 min. The NaClO was washed with sterile water for five times. The residual water on the seeds was dried using sterile filter paper. The sterilized seeds were cultured on induction medium containing 2,4-dichlorophenoxyacetic acid (2,4-D) to induce callus at 28°C with continuous illumination for 10 days. For the callus subculture, the calli induced for 10 days were cultured on the same fresh callus induction medium containing 2,4-D for 15 days. To dissect the effect of 2,4-D on callus induction, the sterilized seeds were cultured on callus induction medium containing 1.5, 2.5, 3.5, 4.5, and 5.5 mg/L 2,4-D at 28°C for 15 days, respectively. The fresh weight and induction rate of callus were calculated. For the effect of cytokinin [kinetin (KT) or zeatin (ZT)] and α-naphthaleneacetic acid (NAA) on callus regeneration, the calli induced for 15 days was transferred to the regeneration medium containing different concentrations of KT or ZT and NAA combinations for 30 days. The callus regeneration efficiency was calculated according to the corresponding formula.

The genetic transformation method of CLCWR was referred to as methods of cultivated rice with some modifications (Hiei and Komari, 2008). Activation of *A. tumefaciens* strain. *A. tumefaciens* strain EHA105 carrying vector was cultured on YEB solid medium (5 g/L beef extract powder, 5 g/L tryptone, 1 g/L yeast extract, 0.5 g/L MgSO_4_.7H_2_O, 5 g/L sucrose, 10 g/L agar, pH 7.0) containing 50 mg/L kanamycin at 28°C for 2–3 days. Single colonies were cultured in 30–50 ml YEB liquid medium at 28°C for 24 h. Preparation of *Agrobacterium* suspension. The bacteria were collected and diluted with AA suspension containing 20 mg/L acetosyringone to OD_600_ ∼ 0.1. The calli subcultured for 12 days was immersed in AA suspension with *A. tumefaciens* containing 20 mg/L acetosyringone and shaken at 28°C, 180 rpm for about 5 min. The excess bacterial solution of callus was absorbed with sterile filter paper. The infected calli were placed on a co-cultivation medium padded with a layer of AA suspension without *A. tumefaciens* at 25°C for 3 days. After co-cultivation, the calli were washed with sterile water for 6–7 times and then rinsed with sterile water containing 400 mg/L carbenicillin, 180 rpm for 15 min. The calli were placed on filter paper to blot the residual water and dried for 30 min. The calli were placed on a selection medium containing 2.5 mg/L 2, 4-D, 30 mg/L *Hygromycin* B, and 400 mg/L carbenicillin and cultured at 28°C under dark conditions. The selection medium was changed every 15 days until fresh resistant calli sprout. Regeneration of *Hyg*-resistant callus. The vigorous *Hyg*-resistant calli were transferred to the regeneration medium containing 2 mg/L ZT and 0.1 mg/L NAA at tissue culture room (28°C, 16 h light/8 h dark) for about 3 weeks until bud sprout. The regenerated buds were transferred to the root induction medium containing 0.135 mg/L NAA to induce root for 14 days. The young seedlings were hardened by opening the lid of the medium and adding sterile watering at 28°C until the leaves and the roots get strong. The transgenic plants were then transplanted into the soil in a greenhouse [28°C, 12.5 h light (3,000–5,000 LX)/11.5 h dark] under normal water and fertilizer management.

### GUS-Staining Assay

The GUS-staining assay was referred to the previous method (Jefferson, 1987). The transformed callus, leaves, or roots were immersed in GUS-staining solution containing 10 mg/mL *X*-Gluc (Sangon Biotech, China), 0.5 mM K_3_Fe (CN)_6_, 0.5 mM K_4_Fe (CN)_6_⋅3H_2_O, 10 mM Na_2_EDTA, 0.2 M NaH_2_PO_4_⋅2H_2_O, 0.2 M Na_2_HPO_4_⋅12H_2_O, 0.1% Triton X-100, 4% methanol at 37°C for 12 h. The callus, leaves, or roots were decolorized sequentially with 30, 40, 50, 60, and 70% ethanol for 1 h, and finally, 70% ethanol was used for complete decolorization. Finally, the callus, leaves, or roots were immersed in Carnot fixed solution for photographic and microscopic observation. The calli selected by *Hygromycin* for 45 days were stained with GUS staining solution.

### Genotyping of Transgenic Plants

For the transgenic plants’ analysis of CLCWR, genomic DNA was isolated from leaves of T_0_ transgenic plants using the Rapid Plant Genomic DNA Isolation Kit (Sangon Biotech, China). Then the *Hygromycin* gene (*hygromycin phosphotransferase*) was detected for each plant using PCR. Fragments containing target sites were amplified by PCR using primers flanking the target sites and further sequenced to identify mutations. Primers for the mutant genotyping were listed in Supplementary Table 1.

### Calculation Formulae


Callusinductionrate=(numberofinducedcalli/numberofculturedmatureseeds)×100%.

Callusbrowningrate=(numberofcallishowingbrowning/numberofsubculturedcalli)×100%.

Regenerationefficiency=(numberofregeneratedplants/numberofcalliusedtoregeneration)×100%.

SelectivefrequencyofHyg-resistantcallus=(numberofHyg-resistantcalli/numberofAgrobacterium-infectedcalli)×100%.

Transformationefficiency=(numberofGUS-stainedcalli/numberofAgrobacterium-infectedcalli)×100%.

Transgenicplantsratio=(numberofPCR-positiveplants/numberofT plants0)×100%.

## Results

### Callus Induction and Regeneration by Using Scutellum Tissue of Embryos in Mature Seeds of Chaling Common Wild Rice

Chaling common wild rice is an important common wild rice germplasm resource in Asia. It has a prostrate growth habit during the vegetative stage (Supplementary Figure 1A). The colors of the mature caryopsis glume and the seed are black brown and dark red, respectively (Supplementary Figure 1B). CLCWR has strong tillering ability and stress tolerance. However, it possesses adverse traits, such as high plant and long awn (Supplementary Figure 1C). To understand the tissue culture characteristics of CLCWR, we attempt to explore its callus induction and regeneration ability.

**FIGURE 1:**
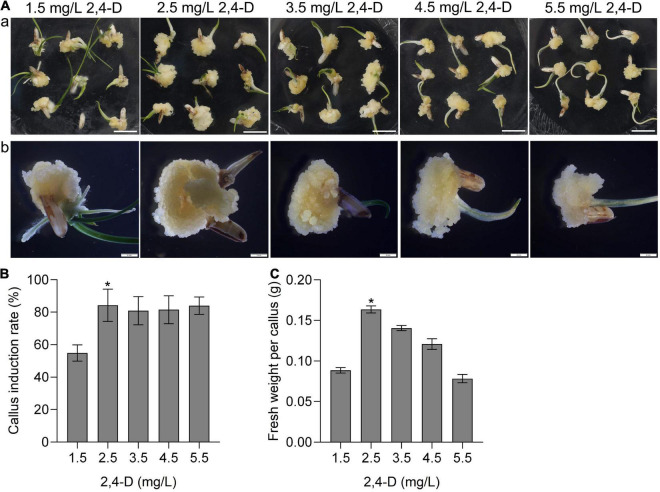
Effects of different concentrations of 2,4-D on calli induction of CLCWR. **(A)** Calli of CLCWR induced on callus induction medium containing different concentrations of 2,4-D. **(a)** Calli were induced on callus induction medium containing 1.5, 2.5, 3.5, 4.5, and 5.5 mg/L of 2,4-D for 15 days by using scutellum tissue of embryos in mature seeds. Bars = 1 cm. **(b)** The calli induced by different concentrations of 2,4-D were enlarged by using the stereomicroscope. Bars = 2 mm. **(B)** Callus induction rate of CLCWR. The callus induction rates were calculated after inducing for 15 days. **(C)** Fresh weight of CLCWR calli referred to **(A)**. Significant differences were found between 2.5 and 1.5, 3.5, 4.5, and 5.5 mg/L of 2, 4-D (*P < 0.01 by Student’s *t-*test). Data are means of three replicates of one experiment. The experiment was repeated three times with similar results. Error bars represent ± SD. Asterisks represent significant differences.

Callus induction is the first step of plant tissue culture. To investigate the callus induction of CLCWR, the scutellum tissue of embryos in mature seeds was cultured on the induction medium with different concentrations of 2,4-D (1.5, 2.5, 3.5, 4.5, and 5.5 mg/L) for 15 days, respectively. The results showed that scutellum tissue of embryos in mature seeds of CLCWR could induce callus under all test concentrations of 2,4-D (Figure 1Aa). The callus induction rate of scutellum tissue of embryos in mature seeds of CLCWR was about 60–90%, and the callus induction rate was highest when the concentration of 2,4-D was 2.5 mg/L (Figure 1B).

The callus texture is one of the important factors affecting the genetic transformation efficiency. We then observed the morphology of callus under different concentrations of 2,4-D culture conditions using the stereomicroscope. The results showed that the size and fresh weight of callus induced by different concentrations of 2,4-D were significantly different (Figure 1Ab), the fresh weight of callus CLCWR were the best when the concentration of 2,4-D was 2.5 mg/L (Figure 1C). These results suggest that callus of CLCWR can be induced by using scutellum tissue of embryos in mature seeds, and 2.5 mg/L concentration of 2,4-D was the best condition for inducing high-quality callus.

**FIGURE 2:**
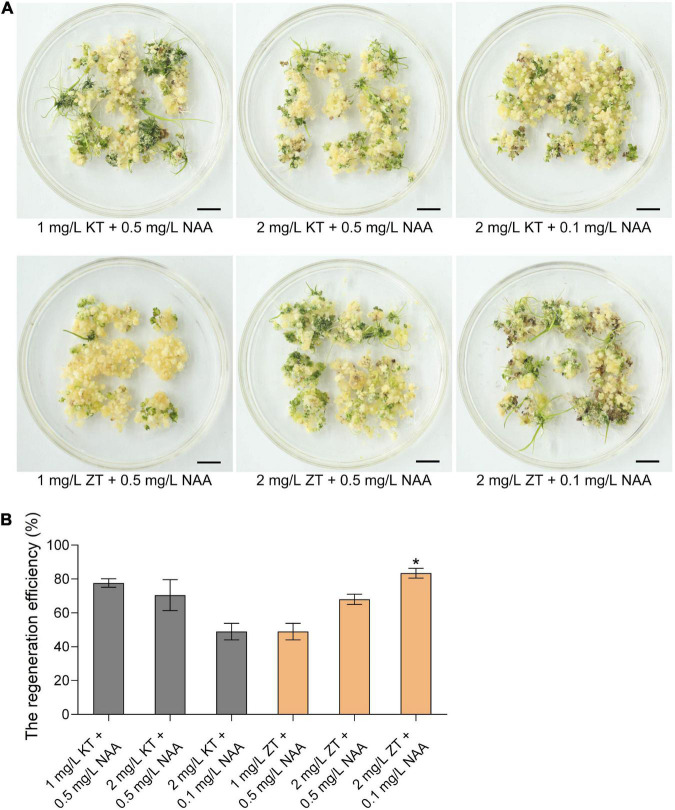
Effects of different combinations of cytokinin and auxin on callus regeneration of CLCWR. **(A)** Calli of CLCWR were induced on callus induction medium containing 2.5 mg/L 2,4-D for 15 days. Then the calli were transferred to the regeneration medium containing different combinations of cytokinin and auxin for 30 days. Bars = 1 cm. **(B)** The regeneration efficiency of calli on the regeneration medium containing different combinations of cytokinin and auxin as indicated in **(A)**. The regeneration efficiency of calli was calculated after regenerating for 30 days. Significant differences were found between 2 mg/L ZT + 0.1 mg/L NAA and other concentration combinations (*P < 0.01 by Student’s *t*-test). Data are means of three replicates of one experiment. The experiment was repeated three times with similar results. Error bars represent ± SD. Asterisks represent significant differences.

Callus regeneration is one of the most critical steps of plant genetic transformation to obtain regenerated plants. To explore the ability of callus regeneration of CLCWR, 15-days old calli were transferred to the regeneration medium with different combinations of cytokinin (KT or ZT) and auxin analog (NAA) for 30 days, respectively. As shown in Figure 2A, the effects of callus regeneration at various concentrations of KT/NAA and ZT/NAA combinations were different. The shoots could be regenerated on all mediums, and the regeneration efficiency was about 50–80%. In addition, the regeneration efficiency of callus was the highest using 2 mg/L ZT + 0.1 mg/L NAA combination (Figure 2B). The above results indicate that the callus of scutellum tissue of embryos in mature seeds of CLCWR has a high regeneration ability.

### Chaling Common Wild Rice Can Be Transformed With High Efficiency Mediated by *Agrobacterium* Infection

**FIGURE 3:**
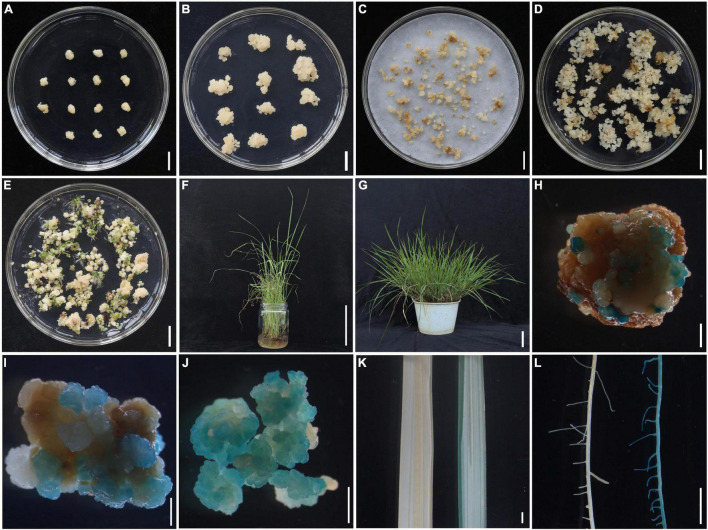
*Agrobacterium*-mediated transformation of CLCWR by using scutellum tissue of embryos in mature seeds. **(A)** Embryogenic calli induced on callus induction medium containing 2.5 mg/L 2,4-D for 10 days. **(B)** Calli subcultured on callus induction medium containing 2.5 mg/L 2,4-D for 12 days. **(C)** Subcultured calli co-cultivated with *Agrobacterium* on co-cultivation medium for 3 days. **(D)** Infected calli were screened by *Hygromycin*. The co-cultured calli were transferred to the selection medium supplemented with 2.5 mg/L 2, 4-D, 30 mg/L *Hygromycin* B, and 400 mg/L carbenicillin for 3 rounds of 15 days each. **(E)** Shoot regeneration from transformed calli. The *Hygromycin*-resistant calli were regenerated on the regeneration medium containing 2 mg/L ZT and 0.1 mg/L NAA for 30 days. **(F)** Roots were induced on the root-induction medium containing 0.135 mg/L NAA for 14 days. **(G)** Transgenic plant after transplanting to the soil for 3 months. **(H–L)** GUS staining of transgenic calli and plant. The calli co-cultivated with *Agrobacterium* **(H)**, selected by *Hygromycin* B **(I)** and regenerated **(J)**, also the leaves **(K)** and roots **(L)** of the transgenic plant (left was non-transgenic and right was transgenic) were stained using GUS staining solution and observed under a stereomicroscope. Scale bars, 1 cm **(A–E)**, 5 cm **(F,G)**, and 2 mm **(H–L)**.

*Agrobacterium*-mediated genetic transformation is the most important technology for the study of gene function in rice. To establish a complete *Agrobacterium*-mediated genetic transformation system under CLCWR background, first, the calli from scutellum tissue of embryos in mature seeds were induced for 10 days (Figure 3A), then subcultured for 12 days to proliferate, the subcultured calli maintained fresh without browning (Figure 3B). Then, the subcultured calli were infected by *Agrobacterium* carrying the recombinant plasmid (pCAMBIA1301-Pro*_*OrNCED*5_*-GUS), and cocultured at 25°C for 3 days in the dark (Figure 3C). The infected calli were washed with sterile water and cultured on the selection medium containing 2.5 mg/L 2, 4-D, 30 mg/L *Hygromycin* B, and 400 mg/L carbenicillin for 3 rounds (a round of 15 days each). We found that a large number of fresh *Hygromycin*-resistant calli pellets were produced, suggesting that callus from scutellum tissue of embryos in mature seeds of CLCWR showed high-transformation efficiency (Figure 3D). Then, we transferred the resistant callus to the regeneration medium for 30 days (Figure 3E). The roots of young seedlings were induced on the root-induction medium supplemented with 0.135 mg/L NAA for 14 days (Figure 3F). Finally, the plants can grow normally when transferred into the soil to grow (Figure 3G).

To investigate whether recombinant plasmid (pCAMBIA1301-Pro*_*OrNCED*5_*-GUS) was transformed into callus of CLCWR, the calli were stained using GUS-staining solution. As shown in Figure 3H, the small and fresh calli pellets cocultured with *Agrobacterium* were dyed blue, indicating that the recombinant plasmid has been successfully transferred into the calli of CLCWR. The 10-days old *Hyg*-resistant calli (Figure 3I) and regenerated calli (Figure 3J) showed the same results. In addition, the leaves (Figure 3K) and roots (Figure 3L) of transgenic plants at the seedling stage were also dyed blue by using the GUS-staining solution. The above mentioned results suggest that the callus from scutellum tissue of embryos in mature seeds of CLCWR can be successfully transformed by *Agrobacterium*.

**FIGURE 4:**
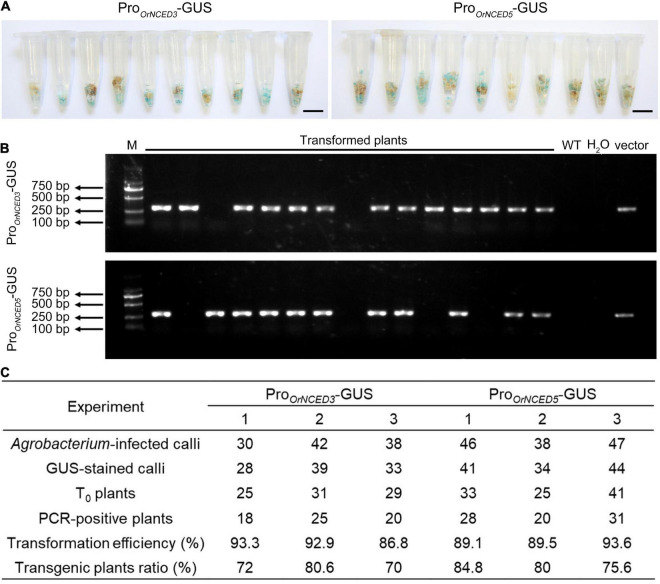
Genetic transformation efficiency of CLCWR. **(A)** GUS staining of transgenic calli selected by *Hygromycin* B for three rounds of 15 d each at 28°C in the dark. Bars = 1 cm. **(B)** Detection of the vector in transformed plants by PCR using *Hyg* gene primer. M, marker, DNA ladder 2000. **(C)** Transformation efficiency and transgenic plants ratio of CLCWR in six experiments. Two GUS reporter vectors were used for transformation. The transformation experiment of each vector was repeated three times. *Agrobacterium*-infected calli, GUS-stained calli, T_0_ plants, and PCR-positive plants were counted in each experiment. Then, the transformation efficiencies and transgenic plants ratios were calculated according to the corresponding formulae.

High-genetic transformation efficiency is an important basis for obtaining positive transgenic plants. To examine the transformation efficiency of CLCWR, 60 independent calli transformed with recombinant plasmids (pCAMBIA1301-Pro*_*OrNCED*3_*-GUS and pCAMBIA1301-Pro*_*OrNCED*5_*-GUS) were used for GUS-staining after selecting by *Hygromycin* for 45 days, respectively. The results showed that about 90% of the *Hyg-*resistant calli could be dyed blue (Figure 4A), suggesting that the transformation efficiency of the callus of CLCWR was high. To further determine the transformation efficiency, 65 independent regenerated plants transformed with pCAMBIA1301-Pro*_*OrNCED*3_*-GUS and pCAMBIA1301-Pro*_*OrNCED*5_*-GUS vectors were used to detect the *Hygromycin phosphotransferase* (*Hyg*) gene by PCR using *Hyg* primers, respectively. Gel electrophoresis showed that the specific ∼280 bp fragments of *Hyg* gene in 60 and 57 plants could be amplified, respectively (Figure 4B), indicating a high proportion of positive plants could be obtained. The above data were displayed that the transformation efficiency of CLCWR was 87–94%, and the transgenic plants ratio was 70–85% (Figure 4C). These results suggest that callus from scutellum tissue of embryos in mature seeds of CLCWR can be transformed with high efficiency mediated by *Agrobacterium* infection.

### Chaling Common Wild Rice Can Be Edited With High Efficiency by CRISPR/Cas9-Mediated Mutagenesis

To explore the genome-editing efficiency under the CLCWR background, the abscisic acid (ABA) synthase genes (*NCED1, NCED3*, and *NCED5*) were selected to edit. The genome-editing plants were generated by using the established genetic transformation system. A total of 60 independent transgenic plants transformed with *NCED3* and *NCED5* single-genome-editing vector were detected by PCR amplification of *Hyg* gene, respectively, both of them showed about 40 positive lines (Supplementary Figure 2). We obtained three types of single-genome-editing lines of *NCED3* (Supplementary Figure 3A) and *NCED5* (Supplementary Figure 3B) by analyzing the target sequences of positive plants, respectively. All of the mutants had a 1-nucleotide insertion at the target site (Supplementary Figures 3A,B). For multiple-genome-editing, we obtained two types of double-gene-editing mutants by editing *NCED1* and *NCED3* (Supplementary Figure 3C). Both *NCED1* and *NCED3* genes in the *nced1nced3* double mutant showed a 1-nucleotide insertion at the target site and no off-targets were found (Supplementary Figure 3C). The statistics of the proportion of genome-editing plants showed that the single and multiplex genome editing efficiency were about 60–70% (Supplementary Figure 3D) and 20–30% (Supplementary Figure 3E), respectively. These results suggest that CLCWR can be edited with high efficiency by CRISPR/Cas9-mediated mutagenesis.

**FIGURE 5:**
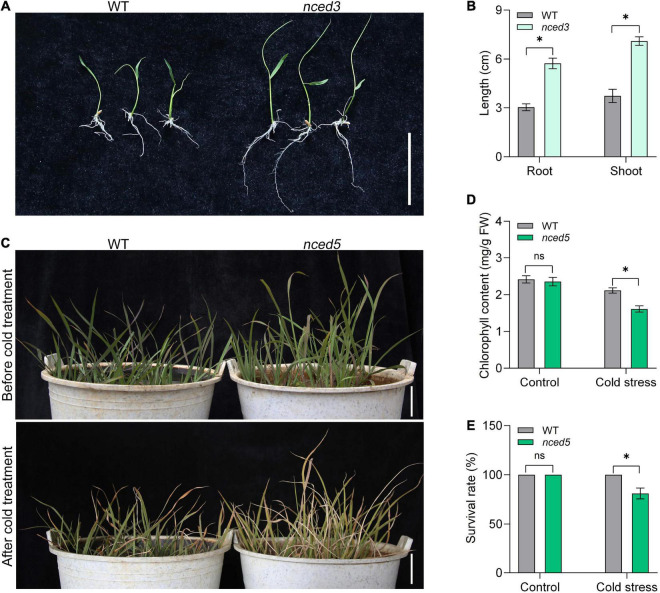
Phenotype analysis of the genome-editing mutants of CLCWR. **(A)** Phenotype analysis of *nced3* mutant. The seeds of wild type and mutant were soaked for 24 h after dormancy was eliminated. Then the seeds were transferred to 37°C incubator for germination for 7 days. Bar = 5 cm. **(B)** The length of root and shoot of wild type and mutant seedlings after germinating for 7 days. **(C)** Phenotype analysis of *nced5* mutant. Wild type and *nced5* mutant seedlings were cultured in the soil until the four-leaf stage. Then the seedlings were treated at 4–6°C for 3 days and then recovered for 7 days. Bars = 5 cm. **(D)** Chlorophyll content of wild type and *nced5* mutant after cold treatment. **(E)** Survival rate of wild type and *nced5* mutant after cold treatment. Significant differences were found between cold stress and control conditions (*P < 0.01 by Student’s *t*-test). Data are means of three replicates of one experiment. The experiment was repeated three times with similar results. Error bars represent ± SD. *, significant; ns, not significant.

To determine whether the inheritance of transgene is stabilized and gene function can be identified under CLCWR background, we then analyzed the phenotypes of *nced3* and *nced5* mutants edited by the CRISPR/Cas9 system. We selected line 1 of *nced3* and *nced5* mutants for further analysis, respectively (Supplementary Figures 3A,B). There was a 1-nucleotide insertion at 430 nucleotides site in *nced3* mutants, resulting in amino acid changes and early termination of translation (Supplementary Figure 4). Similarly, there was a 1-nucleotide insertion at 365 nucleotide sites in *nced5* mutants, resulting in amino acid changes and early termination of translation (Supplementary Figure 4). 9-*cis*-epoxycarotenoid dioxygenase OsNCED3 and OsNCED5 are the key enzymes of ABA biosynthesis and regulate plant growth and abiotic stress tolerance (Huang et al., 2018, 2019). The functional study of *NCED* genes is of great significance for breeding new rice varieties with salt and drought stress tolerance. To determine the function of *NCED3* in CLCWR, the T_1_ seeds of wild type and *nced3* mutants were germinated at 37°C for 7 days after the dormancy was broken (Figure 5A). The results showed that the length of shoot and root of *nced3* mutant was significantly higher than that of wild type (Figure 5B), which was consistent with the results in the Nip background (Huang et al., 2018).

To identify the function of *NCED5* in CLCWR, the *nced5* mutant and wild-type seedlings were treated at 4–6°C for 3 days and then recovered for 7 days (Figure 5C), the results showed that the chlorophyll content and survival rate of *nced5* mutant were significantly lower than that of the wild type after cold stress (Figures 5D,E). The results suggest that inheritance of transgene is stabilized in T_1_ seeds and the function of genes can be identified under the CLCWR background.

### Chaling Common Wild Rice Has Higher Genetic Transformation Efficiency Than Nip

**FIGURE 6:**
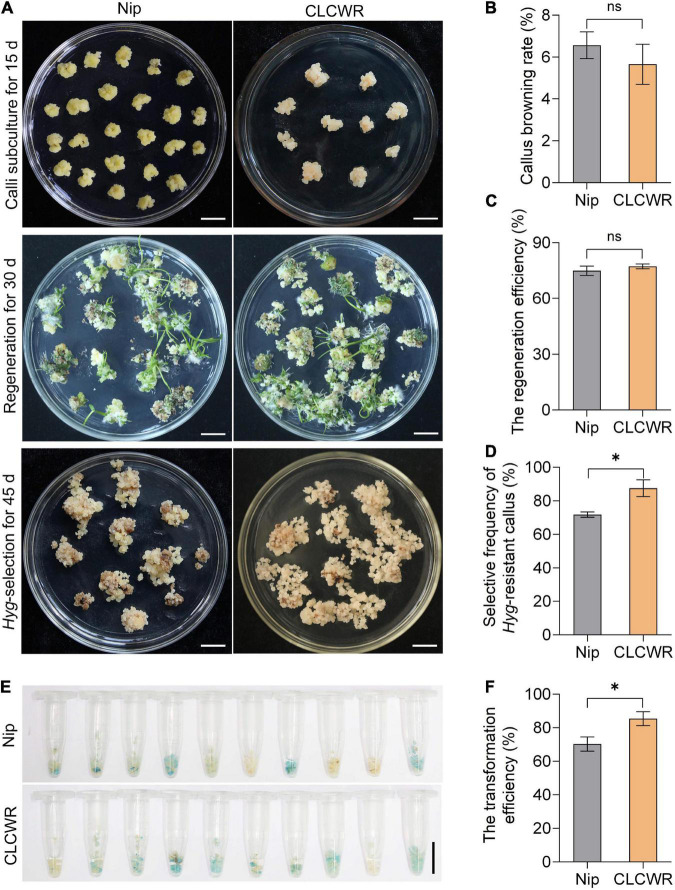
Comparison of *Agrobacterium*-mediated transformation procedure between Nipponbare and CLCWR. **(A)** Callus subculture, regeneration and *Hyg*-resistant selection of Nip and CLCWR. For callus subculture, the calli induced for 10 days were transferred to callus induction medium containing 2, 4-D for 15 days. For regeneration, the fresh calli were cultured on the regeneration medium for 30 days. For *Hyg*-resistant selection, the *Agrobacterium*-infected calli were cultured on a selection medium for three rounds of 15 days each. Bars = 1 cm. **(B–D)** Comparison of the callus browning rate **(B)**, regeneration efficiency **(C)**, and selective frequency of *Hyg*-resistant callus **(D)** between Nip and CLCWR. **(E)** GUS staining of calli transformed with vector pCAMBIA1301-Pro*_*OrNCED*5_*-GUS. **(F)** Comparison of the transformation efficiency between Nip and CLCWR. Significant differences were found between Nip and CLCWR (*P < 0.01 by Student’s *t*-test). Data are means of three replicates of one experiment. The experiment was repeated three times with similar results. Error bars represent ± SD. *, significant; ns, not significant.

Nipponbare is a model variety for the study of gene function in rice and possesses good genetic transformation efficiency. We compared the tissue culturability and genetic transformation between CLCWR and Nip. As shown in Figure 6A, the subcultured calli remained fresh and numerous young seedlings were regenerated in both CLCWR and Nip. The callus browning rate of CLCWR and Nip were both low (Figure 6B) and the regeneration efficiency was both high (Figure 6C). There was no significant difference between the two *Oryza* accessions, indicating that both of them had good tissue culturability. For *Hygromycin* selection process, CLCWR displayed more *Hyg-*resistant calli than Nip (Figure 6A). The selective frequency of the *Hyg*-resistant callus of CLCWR was about 20% higher than that of Nip (Figure 6D). A total of 50 independent *Hyg-*resistant calli were then stained using GUS-staining solution. The callus was about 60 and 80% for Nip and CLCWR were dyed blue, respectively (Figure 6E). The transformation efficiency of CLCWR was about 20% higher than that of Nip (Figure 6F). These results suggest that CLCWR has higher genetic transformation efficiency than Nip.

## Discussion

With the changing environment, cultivating multi-resistant rice is a goal of agriculture development (Fernie and Yan, 2019; Yan et al., 2021). An efficient genetic transformation system was established by using the allotetraploid wild rice PPR1. Hence, rapid *de novo* domestication was realized by editing the genes controlling adverse traits, such as seed shattering, high plant height, and long heading date (Yu et al., 2021). CLCWR seeds can germinate in deep soil under insufficient oxygen and light conditions. Its perennial roots can overwinter safely in the field environment, showing strong cold tolerance (Xu et al., 2020). CLCWR also possesses the advantages of disease and insect resistance and high nutrient utilization efficiency. In the future, CLCWR is used for rapid *de novo* domestication will be of great significance for breeding rice varieties with multi-resistance and high-nutrient utilization efficiency. However, the genetic transformation of CLCWR has not been successfully established. In the present study, we used CLCWR to successfully establish an *Agrobacterium*-mediated genetic transformation system based on scutellum tissue of embryos in mature seeds (Supplementary Figure 5).

The genetic transformation of several wild *Oryza* accessions had been reported, however, its regeneration and transformation efficiency were low (Zhang et al., 2019; Shimizu-Sato et al., 2020; Yu et al., 2021). An efficient genetic transformation system for wild rice is needed to be well established. Although immature embryos can be used to induce callus and are suitable for a broad range of wild *Oryza* accessions, the collection of immature embryos is restricted for growth period and season factors and the operation of acquiring immature embryos is cumbersome (Hiei and Komari, 2008; Shrawat and Good, 2011). The mature seeds can be stored for a long time, and their acquisition is not limited by season, so the operation of the tissue culture process using mature seeds is fast and convenient. In this study, the scutellum tissue of embryos in mature seeds of CLCWR were used for tissue culture and the calli showed good texture (Figure 1), high-regeneration efficiency (Figure 2), and high transformation, and genome-editing efficiency (Figure 4 and Supplementary Figures 3, 5). 2,4-D is an auxin widely used in callus induction. Variant concentrations of 2,4-D are used for callus induction of different genotypic *Oryza* accessions. The concentration of 2,4-D used for callus induction of cultivated rice Nip (*japonica*), Kasalath (*indica*), and common wild rice is 2 mg/L, meanwhile 2.5 mg/L for IR64 (*indica*) (Hiei et al., 1994; Saika and Toki, 2010; Sahoo and Tuteja, 2012; Shimizu-Sato et al., 2020). In this study, 2.5 mg/L 2,4-D was used for CLCWR and shows the best induction efficiency (Figure 1). Cytokinin and auxin are necessary hormones for callus regeneration. The concentration of KT used for cultivated rice is about 1–2 mg/L and the NAA is about 0.2–0.5 mg/L (Hiei et al., 1994; Lin and Zhang, 2005; Saika and Toki, 2010; Sahoo and Tuteja, 2012). The calli of CLCWR could be regenerated by using KT or ZT, and the combination(2.0 mg/L ZT + 0.1 mg/L NAA) was the most suitable combination (Figure 2). Compared with the genetic transformation systems of other wild *Oryza* accessions, we found that ZT has higher regeneration efficiency than that for KT. Compared with the genetic transformation system of PPR1, it took about 120 days to obtain T_0_ transgenic plants, however, which took about 100 days for CLCWR, which markedly reduce the time of tissue culture. Compared with callus-induced material as used in the immature embryos (Shimizu-Sato et al., 2020), we used mature seeds to induce callus as the transformation receptor, which makes the experimental operation fast and convenient. Three hormones (2,4-D, NAA, and 6-BA) were used to induce callus when immature embryos were used for transformation receptors (Shimizu-Sato et al., 2020), however, only one hormone (2,4-D) was used and got the same effect in our study. Taken together, CLCWR possesses high-genetic transformation and genome editing efficiency (Figure 4 and Supplementary Figure 3), which provides the basis for successful *de novo* domestication of CLCWR.

Polyploid rice 1, *O. alta*, an allotetraploid wild rice with a CCDD genome from South America, has large biomass and strong stress resistance. However, PPR1 shows typical non-domesticated features, such as very high plant height (>2.7 m), long awns (>4 cm), and small grain size (Yu et al., 2021). Compared with PPR1, CLCWR from south China is more closely related to cultivated rice, so it may be easy to get rice varieties with excellent traits by using CLCWR for the followed reasons: (1) Both CLCWR and cultivated rice possess the AA genome. Fertile hybrids can be obtained by crossing the *de novo* domesticated CLCWR with diploid cultivated rice, while there may be no offspring when using the PPR1 hybridize with cultivated rice. (2) Compared with PPR1, the plant height of diploid CLCWR is about 1.2 m, which is much shorter than that of PPR1. The 1,000-weight of CLCWR grain (17.73 g) is larger than that of PPR1 (8.79 g). The degree of non-domesticated features in CLCWR is lower than that of PPR1. Therefore, CLCWR may be more suitable used for the improvement of Asian cultivated rice. The release of *indica* and *japonica* rice reference genomes has greatly promoted the research of rice gene function and the molecular breeding process (Goff et al., 2002; Yu et al., 2002; Matsumoto et al., 2016). The high-quality reference genome of allotetraploid wild rice PPR1 was assembled and a large number of excellent genes were discovered (Yu et al., 2021). Therefore, the reference genome is the cornerstone of functional genomics. A high-quality reference genome of CLCWR is expected to be sequenced and assembled, which could be used to promote molecular design breeding by using CLCWR in the future.

## Data Availability Statement

The datasets presented in this study can be found in online repositories. The names of the repository/repositories and accession number(s) can be found in the article/Supplementary Material.

## Author Contributions

ZX, DM, and LC conceived and designed the experiments and analyzed the data. ZX, YiC, YaC, LZ, and ML performed the experiments. ZX and DM wrote the manuscript. All authors contributed to the article and approved the submitted version.

## Conflict of Interest

The authors declare that the research was conducted in the absence of any commercial or financial relationships that could be construed as a potential conflict of interest.

## Publisher’s Note

All claims expressed in this article are solely those of the authors and do not necessarily represent those of their affiliated organizations, or those of the publisher, the editors and the reviewers. Any product that may be evaluated in this article, or claim that may be made by its manufacturer, is not guaranteed or endorsed by the publisher.

## Supplementary Material

Supplementary Figure 1 Agronomic traits analysis of CLCWR. **(A)** The CLCWR plant cultured in soil for 180 days. Bar = 10 cm. **(B)** The mature seeds of CLCWR. Bar = 1 cm. **(C)** Agronomic traits of CLCWR. Data are means of three replicates of one experiment. The experiment was repeated three times with similar results.

Supplementary Figure 2 PCR verified of the positive transgenic plants under CLCWR background. The positive transgenic plants of CLCWR transformed with single-genome-editing vector were detected by PCR using *Hyg* gene primer. M, marker.

Supplementary Figure 3 Genome editing of CLCWR. **(A,B)** Single genome editing by using CRISPR/Cas9 multiplex genome editing system. *OrNCED3* **(A)** and *OrNCED5* **(B)** were used for single genome editing analysis and produced three types of mutants, respectively. **(C)** Two-genome editing by using CRISPR/Cas9 multiplex genome editing system. *OrNCED1* and *OrNCED3* were used for multiplex genome editing analysis and produced two types of mutants. **(D–E)** Genome-editing efficiency of CLCWR. A total of 20 PCR-positive plants were used for sequencing analysis in each experiment. The sequences of the edited genes of PCR-positive plants were compared by DNAMAN software and analyzed by DSDecode M (http://www.ygliulab.club/dsdecode/). Then, the number of edited plants was counted. The single **(D)** and multiplex **(E)** genome editing efficiencies were calculated as the number of mutated plants divided by the number of PCR-positive plants.

Supplementary Figure 4 Amino acid sequence comparison of mutants and WT by DNAMAN software. **(A)** Amino acid sequence comparison of *nced3* mutants and WT. **(B)** Amino acid sequence comparison of *nced5* mutants and WT.

Supplementary Figure 5 Schematic of *Agrobacterium*-mediated genetic transformation system of CLCWR. Schematic of *Agrobacterium*-mediated genetic transformation system of CLCWR using scutellum tissue of embryos in the mature seeds.

Supplementary Table 1 Primers used in this study.

Supplementary Table 2 The culture medium used in this study.
