# Supplementary figures and images for: Agrobacterium-Mediated High-Efficiency Genetic Transformation and Genome Editing of Chaling Common Wild Rice (Oryza rufipogon Griff.) Using Scutellum Tissue of Embryos in Mature Seeds
Source: Front Plant Sci. 2022 Mar 24;13:849666. doi: 10.3389/fpls.2022.849666 (PMC8988072; doi:10.3389/fpls.2022.849666)

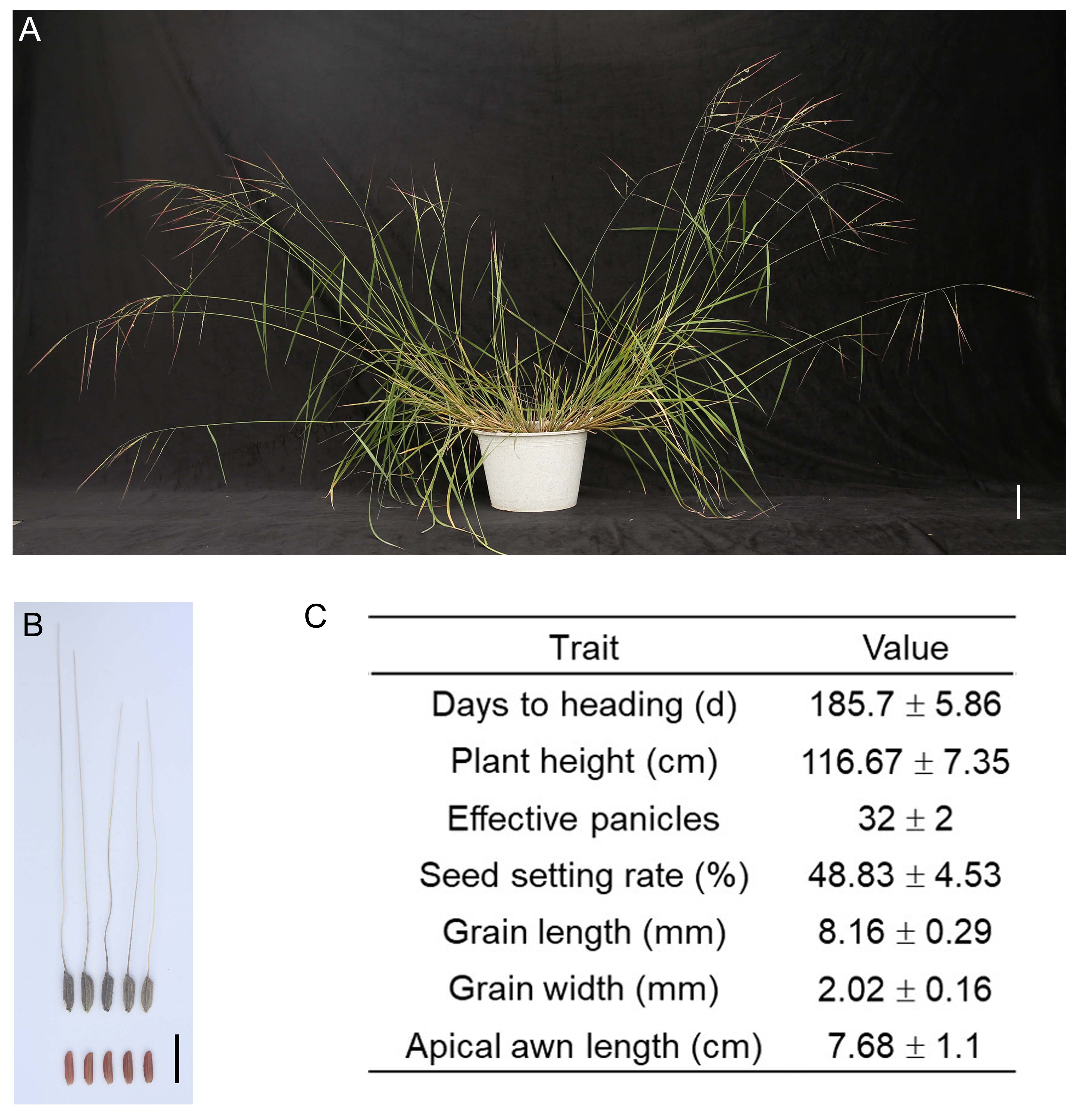

Supplement: Supplementary Figure 1 — Agronomic traits analysis of CLCWR. (A) The CLCWR plant cultured in soil for 180 days. Bar = 10 cm. (B) The mature seeds of CLCWR. Bar = 1 cm. (C) Agronomic traits of CLCWR. Data are means of three replicates of one experiment. The experiment was repeated three times with similar results. [file Image_1.JPEG]

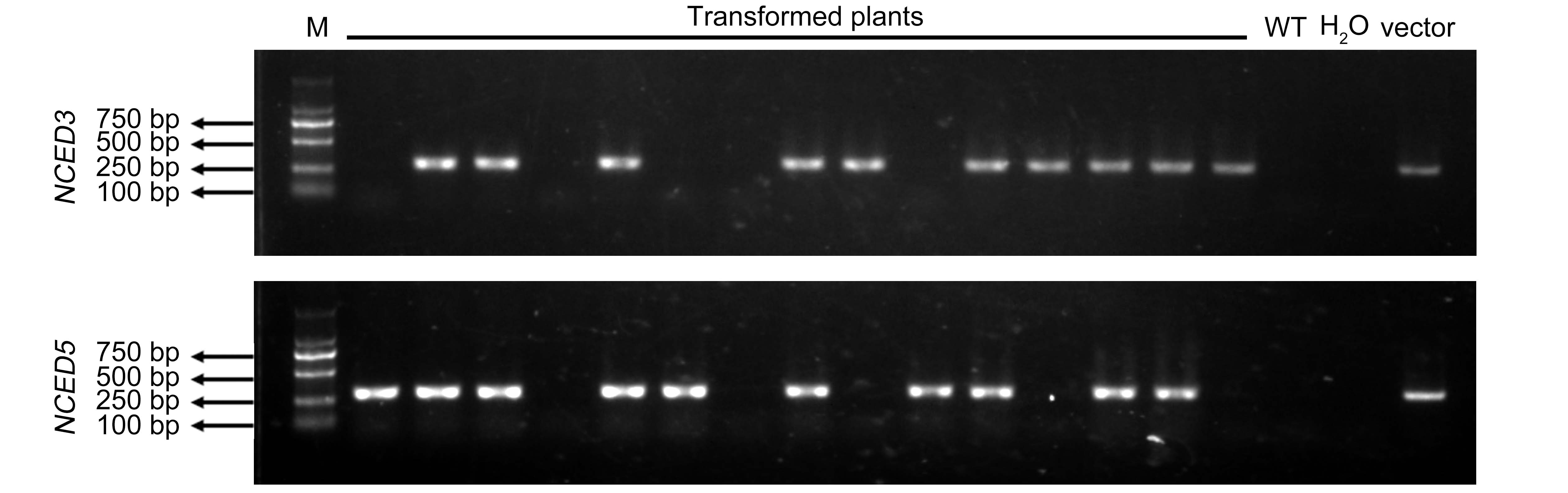

Supplement: Supplementary Figure 2 — PCR verified of the positive transgenic plants under CLCWR background. The positive transgenic plants of CLCWR transformed with single-genome-editing vector were detected by PCR using Hyg gene primer. M, marker. [file Image_2.JPEG]

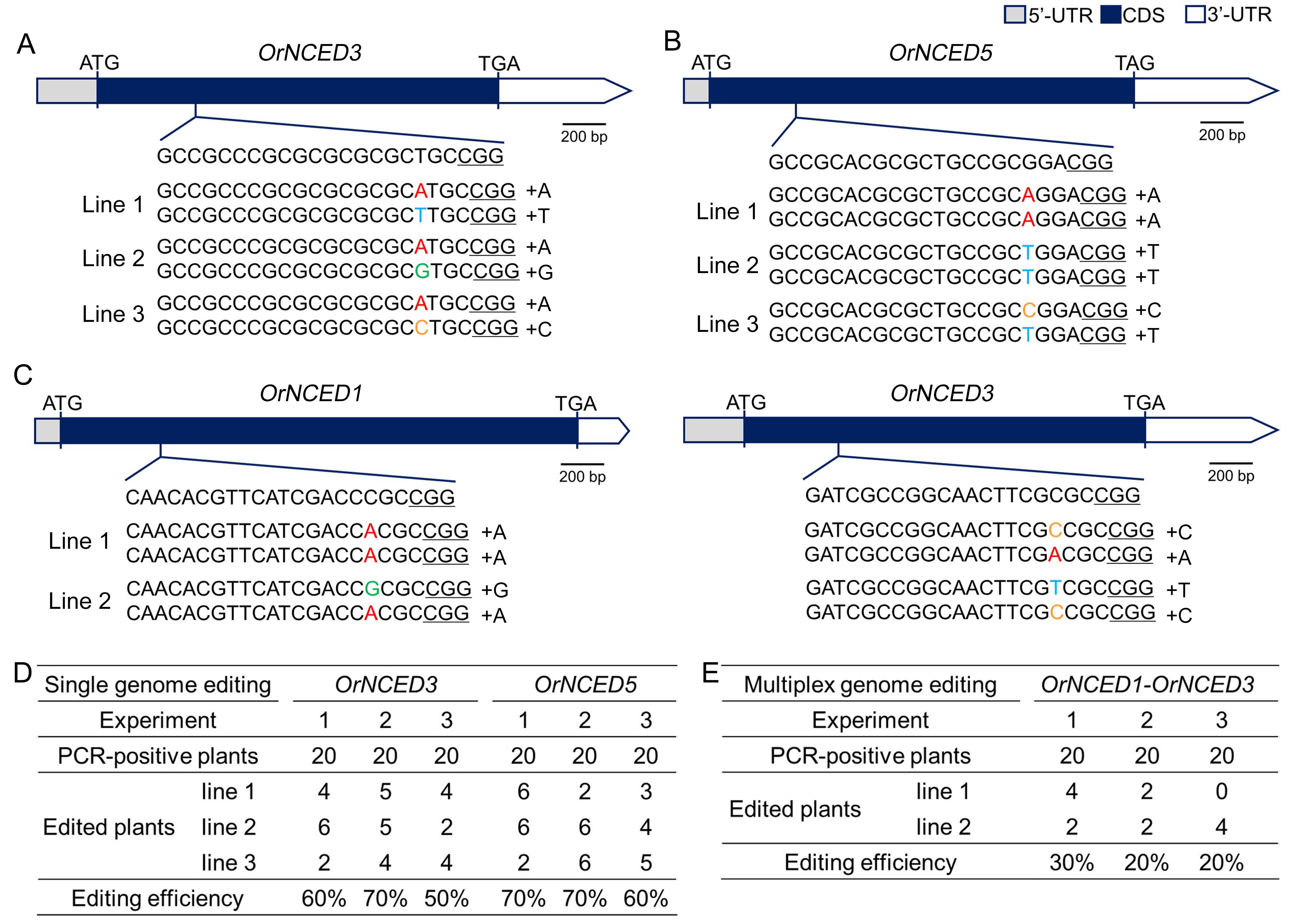

Supplement: Supplementary Figure 3 — Genome editing of CLCWR. (A,B) Single genome editing by using CRISPR/Cas9 multiplex genome editing system. OrNCED3 (A) and OrNCED5 (B) were used for single genome editing analysis and produced three types of mutants, respectively. (C) Two-genome editing by using CRISPR/Cas9 multiplex genome editing system. OrNCED1 and OrNCED3 were used for multiplex genome editing analysis and produced two types of mutants. (D–E) Genome-editing efficiency of CLCWR. A total of 20 PCR-positive plants were used for sequencing analysis in each experiment. The sequences of the edited genes of PCR-positive plants were compared by DNAMAN software and analyzed by DSDecode M (http://www.ygliulab.club/dsdecode/). Then, the number of edited plants was counted. The single (D) and multiplex (E) genome editing efficiencies were calculated as the number of mutated plants divided by the number of PCR-positive plants. [file Image_3.JPEG]

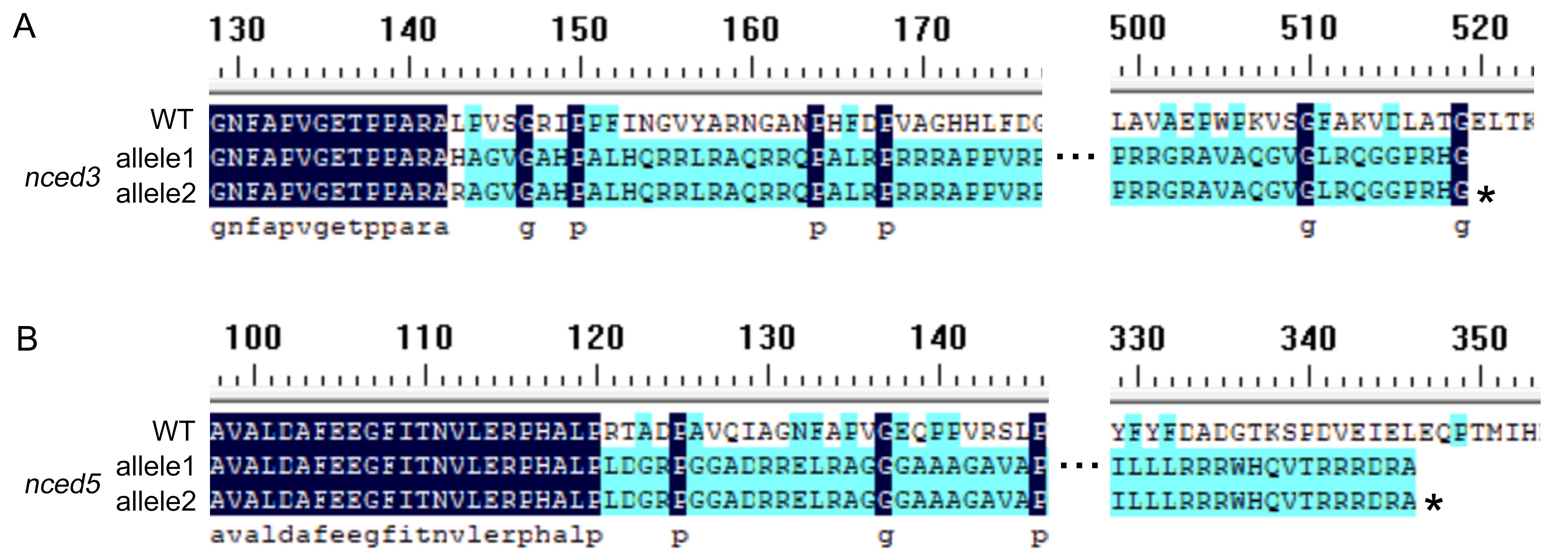

Supplement: Supplementary Figure 4 — Amino acid sequence comparison of mutants and WT by DNAMAN software. (A) Amino acid sequence comparison of nced3 mutants and WT. (B) Amino acid sequence comparison of nced5 mutants and WT. [file Image_4.JPEG]

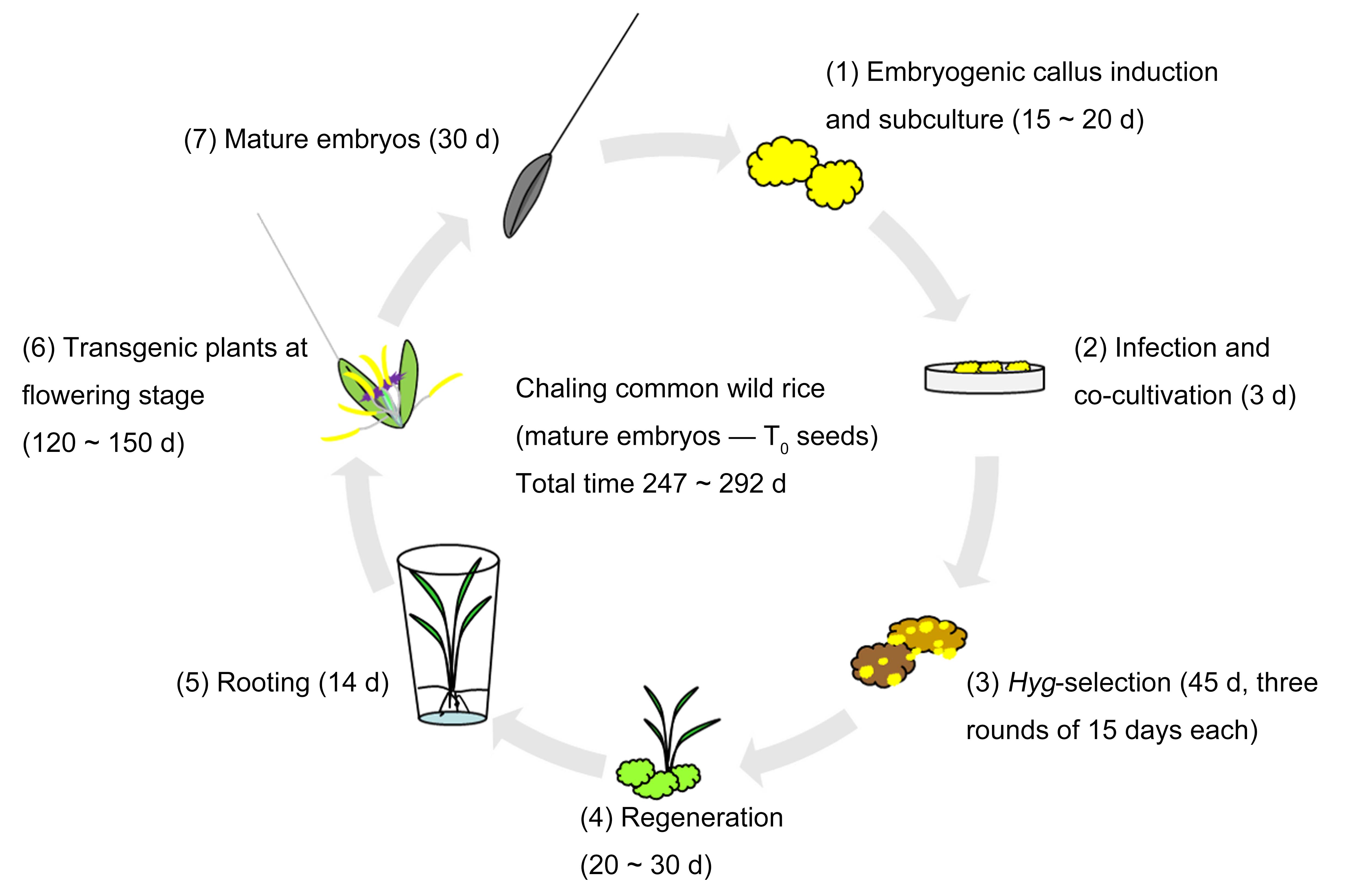

Supplement: Supplementary Figure 5 — Schematic of Agrobacterium-mediated genetic transformation system of CLCWR. Schematic of Agrobacterium-mediated genetic transformation system of CLCWR using scutellum tissue of embryos in the mature seeds. [file Image_5.JPEG]
