# Supplementary material for: Agrobacterium-Mediated High-Efficiency Genetic Transformation and Genome Editing of Chaling Common Wild Rice (Oryza rufipogon Griff.) Using Scutellum Tissue of Embryos in Mature Seeds
Source: Front Plant Sci. 2022 Mar 24;13:849666. doi: 10.3389/fpls.2022.849666 (PMC8988072; doi:10.3389/fpls.2022.849666)
Supplement: Supplementary Table 1 — Primers used in this study. [file Data_Sheet_1.PDF]

Supplemental Table 1. Primers used in this study.

Primer	Name	Forward 5'-3'	Reverse 5'-3'
CDS amplification	*OrNCED1*-CDS	ATGCAAAGGATTTGCCCTGC	TTGGTGCTGTGACTGGAGCTC
	OrNCED3-CDS	ATGGCGACGATCACGACG	GGCCTGGGTGGTGAGCTC
	OrNCED5-CDS	ATGCCGACCACCTTCACGC	GGCCTGCGTGGCGAGCTC
Genome editing	*OrNCED1*-cas9	CAACACGTTCATCGACCCGC	GCGGGTCGATGAACGTGTTG
		gttttagagctagaaat	cggcagccaagccagca
	OrNCED3-cas9-1	CCGCCCGCGCGCGCGCTGC	GCAGCGCGCGCGCGGGCGG
		gttttagagctagaaat	cggcagccaagccagca
	OrNCED3-cas9-2	ATCGCCGGCAACTTCGCGC	GCGCGAAGTTGCCGGCGAT
		gttttagagctagaaat	caacacaagcggcagc
	OrNCED5-cas9	CCGCACGCGCTGCCGCGGA	TCCGCGGCAGCGCGTGCGG
		gttttagagctagaaat	cggcagccaagccagca
Promoter amplification	*OrNCED3*-Pro	tatgaccatgattacgaattcCCCTTTGATCAGCAT	acgacggccagtgccaagcttATCGCGTCGATCGC
		GTTAAACAT	ACAA
	OrNCED5-Pro	tatgaccatgattacgaattcCATCAACTAAGAGCT	acgacggccagtgccaagcttGGCTGTCCTACAAG
		CAACGAGTACTC	AGCAGAACA
Mutation genotyping	*OsNCED1*-JC	ATAGCCGCACGGTTAATCTCA	AAGCCGGAGAAGACGTTGG
	OsNCED3-JC	TCCCAAACCATCCAAACCG	GCCGTTGCGTATCCTGACG
	OsNCED5-JC	CTCCTCTTCTCCCGCCAACT	GCCGTTGAAGTAGACGAGGC
Hyg gene detection	*Hygromycin* (*hyg*)	ACGGTGTCGTCCATCACAGTTTGCC	TTCCGGAAGTGCTTGACATTGGGGA

Supplemental Table 2. Culture medium used in this study.

Medium	Components (1 L)
Callus induction (pH 5.8)	N_6 Macronutrient[a]: 2830 mg KNO_3, 463 mg $(NH_4)_2SO_4$, 400 mg KH_2PO_4, 185 mg $MgSO_4 \cdot 7H_2O$, 166 mg $CaCl_2 \cdot 2H_2O$<br>B_5 Micronutrient[a]: 27.8 mg $FeSO_4 \cdot 7H_2O$, 37.3 mg Na_2EDTA, 10 mg $MnSO_4 \cdot 4H_2O$, 2 mg $ZnSO_4 \cdot 7H_2O$, 3 mg H_3BO_3, 0.75 mg KI, 0.025 mg $CuSO_4 \cdot 5H_2O$, 0.25 mg $Na_2MoO_4 \cdot 2H_2O$, 0.025 mg $CoCl_2 \cdot 6H_2O$<br>Organics: 2 mg glycine, 1 mg thiamine, 0.5 mg pyridoxine, 0.5 mg nicotinic acid, 100 mg *myo*-inositol, 2878 mg proline, 500 mg L-glutamine, 600 mg casin hydrolysate, 30000 mg sucrose, 4600 mg phytagel (Sigma)<br>Plant hormone: 2,4-D (1.5, 2.5, 3.5, 4.5 and 5.5 mg)
AA (pH 5.2)	Macronutrient: 2950 mg KCl, 170 mg Na_2HPO_4, 500 mg $MgSO_4 \cdot 7H_2O$, 150 mg $CaCl_2 \cdot 2H_2O$<br>27.8 mg $FeSO_4 \cdot 7H_2O$, 37.3 mg Na_2EDTA, 10 mg $MnSO_4 \cdot 4H_2O$, 2 mg $ZnSO_4 \cdot 7H_2O$, 3 mg H_3BO_3, 0.75 mg KI, 0.025 mg $CuSO_4 \cdot 5H_2O$, 0.25 mg $Na_2MoO_4 \cdot 2H_2O$, 0.025 mg $CoCl_2 \cdot 6H_2O$<br>Organics: 2 mg glycine, 10 mg thiamine, 1 mg pyridoxine, 1 mg nicotinic acid, 100 mg *myo*-inositol, 876 mg L-glutamine, 500 mg casin hydrolysate, 68500 mg sucrose, 36000 mg glucose, 216 mg L-asparagine, 176 mg L-arginine, 20 mg acetosyringone(Sangon Biotech, China)
Co-cultivation (pH 5.8)	N_6 Macronutrient + B_5 Micronutrient<br>Organics: 2 mg glycine, 1 mg thiamine, 0.5 mg pyridoxine, 0.5 mg nicotinic acid, 100 mg *myo*-inositol, 500 mg L-glutamine, 600 mg casin hydrolysate, 30000 mgsucrose, 10000 mg glucose, 20 mg acetosyringone, 4600 mg phytagel<br>2.5 mg 2,4-D
*S*election (pH 5.8)	N_6 Macronutrient + B_5 Micronutrient + Callus induction organics<br>Plant hormone: 2.5 mg 2,4-D<br>*Hygromycin*B (Invitrogen, USA): 30 mg<br>Carbenicillin(Sangon Biotech, China): 400 mg
Regeneration (pH 5.8)	MSMacronutrient: 1900 mg KNO_3, 1650 mg NH_4NO_3, 170 mg KH_2PO_4, 370 mg $MgSO_4 \cdot 7H_2O$, 440 mg $CaCl_2 \cdot 2H_2O$<br>MSMicronutrient: 27.85 mg $FeSO_4 \cdot 7H_2O$, 37.25 mg Na_2EDTA, 22.3 mg $MnSO_4 \cdot 4H_2O$, 8.6 mg $ZnSO_4 \cdot 7H_2O$, 6.2 mg H_3BO_3, 0.83 mg KI, 0.025 mg $CuSO_4 \cdot 5H_2O$, 0.25 mg $Na_2MoO_4 \cdot 2H_2O$, 0.025 mg $CoCl_2 \cdot 6H_2O$<br>Organics: 2 mg glycine, 0.4 mg thiamine, 0.5 mg pyridoxine, 0.5 mg nicotinic acid, 100 mg *myo*-inositol, 2878 mg proline, 2000 mg casin hydrolysate, 30000 mg sucrose, 30000 mg D-sorbitol, 4200 mg phytagel<br>Plant hormones[b]: 2 mg ZT, 0.1 mg NAA
Root-induction (pH 5.8)	MS Macronutrient + MS Micronutrient<br>Organics: 2 mg glycine, 0.4 mg thiamine, 0.5 mg pyridoxine, 0.5 mg nicotinic acid, 100 mg *myo*-inositol, 2000 mg casin hydrolysate, 30000 mg sucrose, 4200 mg phytagel<br>Plant hormone: 0.135 mg NAA

[a] N_6 Macronutrient, B_5 Micronutrient componentsreferred to Linand Zhang (2005).

[b] For effect of KT or ZT, different concentrations of KT or ZT and NAA combinations weredesigned: 1 mg/L KT + 0.5 mg/L NAA, 2 mg/L KT + 0.1 mg/L NAA, 2 mg/L KT + 0.5 mg/L NAA, 1 mg/L ZT + 0.5 mg/L NAA, 2 mg/L ZT + 0.1 mg/L NAA, 2 mg/L ZT + 0.5 mg/L NAA.
